# INFLUENCE OF DILUTION, TIME, AND TEMPERATURE AFTER PREPARATION ON THE OSMOLALITY OF INFANT FORMULAS GIVEN TO NEWBORNS

**DOI:** 10.1590/1984-0462/;2018;36;4;00009

**Published:** 2018

**Authors:** Isabella Nascimento Alves Ferreira, Fernanda Valente Mendes Soares, Ana Carolina Carioca da Costa, Maria Elisabeth Lopes Moreira

**Affiliations:** a Fundação Oswaldo Cruz, Rio de Janeiro, RJ, Brasil.

**Keywords:** Infant formulas, Osmolality, Food security, Fórmulas infantis, Osmolalidade, Segurança alimentar

## Abstract

**Objective:**

To analyze the influence of dilution, time, and temperature after preparation on the osmolality of infant formulas given to newborns (NBs).

**Methods:**

Experimental and descriptive study with samples of different neonatal formulas to verify the osmolality of the milk according to dilution, time, and temperature after preparation. We analyzed seven neonatal formulas in the following times after preparation: immediately (up to 5 minutes); 20 and 40 minutes; every hour up to 8 hours; and 12 and 24 hours. The samples were evaluated at room temperature and after refrigeration. Osmolality curves were designed with the mean of triplicate samples of each milk sample. The digital Osmometer A+, model 3320, from Advanced Instruments measured the osmolality.

**Results:**

The time and temperature at which the milk was subjected after preparation did not cause the osmolality to exceed its safety cut-off point at a 1:30 dilution in any of the types of milk analyzed. At a 1:25 dilution, the formula with prebiotics in its composition went over the limit after 4 hours.

**Conclusions:**

The milk tested did not exceed the cut-off point of 450 mOsm/kg (approximately 400 mOsm/L), indicated as safe by the American Academy of Pediatrics (AAP) at a dilution recommended by manufacturers. It is important to know the factors that may or may not contribute to the rise of osmolality, in order to establish safe and quality practices for NBs, following protocols based on scientific evidence.

## RESUMO

**Objetivo:**

Analisar a influência da diluição, o tempo e a temperatura pós-preparo na osmolalidade das fórmulas lácteas oferecidas a recém-nascidos (RN).

**Métodos:**

Estudo experimental e descritivo com uma amostra de diferentes fórmulas lácteas neonatais para verificar a osmolalidade do leite segundo a diluição, o tempo e a temperatura pós-preparo. Foram analisadas sete fórmulas lácteas neonatais nos seguintes tempos: imediatamente (até 5 minutos) após o preparo; 20 e 40 minutos após o preparo; de hora em hora, até completar 8 h; e após 12 e 24 h de preparo. As amostras foram avaliadas após o preparo em temperatura ambiente e depois da refrigeração. Foram elaboradas curvas de osmolalidade com a média das triplicatas de cada amostra de leite. O cálculo da osmolalidade foi aferido no Osmômetro digital A+ da Advanced Instruments, modelo 3.320.

**Resultados:**

O tempo e a temperatura em que os leites ficaram submetidos após o preparo não ultrapassaram o ponto de corte de segurança da osmolalidade na diluição 1:30 em nenhum dos tipos de leite analisados. Na diluição 1:25 a fórmula láctea que possui prebióticos em sua composição ultrapassou o limite após 4 h de preparo.

**Conclusões:**

Os leites testados não ultrapassaram o ponto de corte de 450 mOsm/kg (aproximadamente 400 mOsm/L), indicado como seguro pela Academia Americana de Pediatria (AAP) na diluição preconizada pelos fabricantes. É necessário conhecer os fatores que podem ou não contribuir com o aumento da osmolalidade, afim de se estabelecer condutas seguras e de qualidade para os RN, seguindo protocolos baseados em evidências científicas.

## INTRODUCTION

Nutrition in early life is currently recognized as a determinant factor for the improvement of neonatal results in preterm newborns (PTNBs).^1^ Breast milk (BM) is considered the first and ideal food choice for full-term and preterm newborns (NBs), due to its nutritional and immunological components.^2^ If giving BM, with or without the use of additives, is not possible, infant formulas are indicated to meet the clinical and nutritional needs of PTNBs. However, powdered infant formulas can become unsafe for NBs if the preparation is not adequate, mainly with respect to dilution, which can modify the osmolality.^3^

The American Academy of Pediatrics (AAP) recommends, for infant formulas, an osmolality cut-off point of 450 mOsm/kg (approximately 400 mOsm/L),^4^ as higher values have been associated with nausea, vomiting, diarrhea, and gastroesophageal reflux, in addition to being closely related to the development of necrotizing enterocolitis (NEC), which is the most severe gastrointestinal complication for extremely low birth weight PTNBs and can lead to death.^5^ ^,^ ^6^ ^,^ ^7^ ^,^ ^8^

Studies describing factors associated with changes in osmolality, such as refrigeration,^9^ acidity,^10^ use of additives,^11^ ^,^ ^12^ and addition of vitamins,^13^ are aimed at BM. The objective of this work was to verify the influence of dilution, refrigeration, and time between preparation and administration on the osmolality of infant formulas given to NBs.

## METHOD

This is an experimental study with samples of infant formulas available on the Brazilian market given to NBs with different dilution rates, time, and temperature between preparation and administration.

The neonatal formulas evaluated were: PreNAN^®^ and NAN 1^®^ (Nestlé Brasil Ltda.); Aptamil Pre^®^ and Aptamil 1^®^ (Danone); Enfamil EnfaCare^®^ and Enfamil 1^®^ (MeadJohnson); and Similac 1^®^ (Abbott). Table 1 presents the nutritional composition and osmolality provided by manufacturers. For ethical reasons, we codified the neonatal formulas in the results.

**Table 1:** Osmolality, macronutrients, and energy values of infant formulas per 100 g of powder as described on the manufacturer’s website.

	PreNAN^®^	NAN 1^®^	Aptamil Pre^®^	Aptamil 1^®^	Enfamil EnfaCare^®^	Enfamil 1^®^	Similac 1^®^
Osmolality (mOsm/kg)	320.00	290.00	330.00	250.00	310.00	-*	303.00
Carbohydrate (g)	53.60	57.90	49.70	53.10	52.00	55.00	55.00
Protein (g)	14.50	11.40	15.40	9.76	14.00	11.00	11.00
Lipids (g)	26.00	25.80	28.30	25.90	27.00	29.00	28.00
Energy value (kcal)	501.00	509.00	514.00	484.00	503.00	523.00	513.00

*Manufacturer 1 did not provide the osmolality value in its website.

The formulas were prepared at 1:30 and 1:25 dilution rates, that is, a measuring scoop for every 30 mL of water and one measuring scoop for every 25 mL of water, respectively. The weight of the powder in the measuring scoop ranged from 4.3 to 5.5 g, according to each manufacturer.

We analyzed the samples immediately (up to 5 minutes) after preparation; 20 and 40 minutes later; every hour up to 8 hours; and after 12 and 24 hours. The samples were evaluated after being kept at room temperature - the temperature of the Laboratory for Quality Control of the Milk Bank of the Instituto Nacional de Saúde da Mulher, da Criança e do Adolescente Fernandes Figueira (IFF), with air-conditioning between 15 and 20ºC - and refrigerated - fridge with control of maximum acceptable temperature of 5ºC.

Osmolality is the number of osmotically active particles present in 1 kg of solvent,^14^ and we used the digital Osmometer A+, 3,320 model (Advanced Instruments, Norwood, United States) to measure it. Thirteen samples of each type of milk from the same batch were analyzed and prepared according to manufacturer’s specifications. We measured the samples in precision scales and analyzed them in triplicate. The results were printed by the printer attached to the equipment and transcribed into a casebook specific for the study, allowing the traceability of data.

We recorded the study results in a database in Microsoft Excel. Osmolality curves were designed with the mean of each triplicate, divided into two sample groups (preterm and full-term infant formula), according to time after preparation and temperature (refrigerated and room temperature). The osmolality cut-off point established for patient safety was 450 mOsm/kg. In addition, Table 2 presents the results through means and their standard deviation. The software used to elaborate the osmolality curves was R version 3.2.2 (Vienna, Austria).^15^

**Table 2:** Mean osmolality, and standard deviation of the formulas analyzed according to stored time and temperature after preparation.

Time	Room temperature		Refrigerated	
	1:30	1:25	1:30	1:25
5 minutes				
Formula for PTNBs 1	342.7±7.2	422.0±21.6	332.7±0.6	426.0±7.0
Formula for PTNBs 2	284.0±9.5	324.3±2.1	280.0±8.7	331.7±4.7
Formula for PTNBs 3	308.7±8.3	373.0±17.3	307.7±0.6	361.7±3.2
Formula for full-term NBs 1	374.6±2.9	440.7±3.5	358.7±3.8	436.0±3.6
Formula for full-term NBs 2	275.3±0.6	367.0±1.7	305.3±1.5	378.0±2.6
Formula for full-term NBs 3	219.7±0.6	311.7±2.9	239.7±1.1	329.3±3.0
Formula for full-term NBs 4	311.3±2.1	372.0±6.9	373.7±1.5	373.7±1.5
1 hour				
Formula for PTNBs 1	351.3±6.5	414.0±7.0	437.0±3.6	373.7±1.5
Formula for PTNBs 2	285.0±4.4	336.0±10.1	272.0±1.5	341.0±12.8
Formula for PTNBs 3	305.7±1.2	363.7±2.1	313.0±7.0	362.7±0.6
Formula for full-term NBs 1	380.3±1.5	445.3±6.6	363.0±1.0	439.7±3.5
Formula for full-term NBs 2	280.3±3.2	377.0±6.0	313.7±1.1	385.0±1.7
Formula for full-term NBs 3	223.0±2.0	315.3±1.5	243.3±2.3	332.7±3.0
Formula for full-term NBs 4	312.7±3.0	369.0±1.0	321.0±3.6	372.7±2.3
6 hours				
Formula for PTNBs 1	364.0±14.1	433.3±12.5	351.3±11.0	439.7±11.5
Formula for PTNBs 2	301.3±9.1	343.3±6.8	281.0±10.4	345.7±6.8
Formula for PTNBs 3	308.0±1.7	371.0±1.7	317.7±9.9	371.7±3.2
Formula for full-term NBs 1	392.7±4.0	459.0±6.2	372.0±1.0	456.3±6.1
Formula for full-term NBs 2	289.0±0.0	392.7±4.7	321.6±4.9	397.3±3.2
Formula for full-term NBs 3	226.0±1.0	392.7±4.7	247.0±1.0	397.3±3.2
Formula for full-term NBs 4	316.3±2.0	378.0±5.2	322.0±1.0	378.7±1.1
12 hours				
Formula for PTNBs 1	361.0±15.5	431.7±10.2	351.3±6.5	449.7±16.6
Formula for PTNBs 2	297.0±0.0	349.7±6.8	282.3±4.9	343.3±2.3
Formula for PTNBs 3	319.0±7.0	386.7±11.7	318.3±7.6	372.7±4.6
Formula for full-term NBs 1	394.3±4.1	464.7±3.2	372.0±1.0	461.0±7.8
Formula for full-term NBs 2	296.7±1.5	402.3±1.1	322.0±1.0	402.7±2.1
Formula for full-term NBs 3	232.7±2.3	331.3±4.9	250.7±1.5	346.0±2.6
Formula for full-term NBs 4	317.7±2.8	376.0±1.0	325.0±1.7	382.0±2.6
24 hours				
Formula for PTNBs 1	363.0±5.2	452.7±19.4	356.0±5.3	455.7±7.0
Formula for PTNBs 2	307.3±4.9	358.0±18.2	286.0±1.7	358.0±12.8
Formula for PTNBs 3	325.3±4.5	386.0±0.0	320.7±4.2	375.3±3.2
Formula for full-term NBs 1	408.7±2.3	490.0±8.2	386.3±2.0	476.3±6.8
Formula for full-term NBs 2	305.0±1.0	413.7±3.7	327.7±2.0	407.3±0.6
Formula for full-term NBs 3	235.7±2.9	331.0±1.0	259.0±6.6	350.0±2.0
Formula for full-term NBs 4	320.7±0.6	384.3±5.0	328.0±1.0	384.0±1.0

PTNBs: preterm newborns; NBs: newborns.

## RESULTS

Table 2 displays the mean osmolality of the formulas analyzed according to storage time and temperature after preparation.

**Figure 1:**
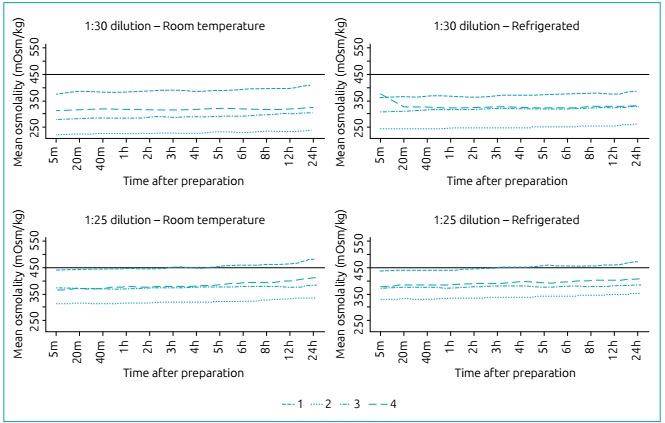
Osmolality analysis according to refrigeration and time between preparation and administration of infant formulas on the market for full-term newborns at 1:30 and 1:25 dilution rates.

Figure 1 shows the osmolality curves according to refrigeration and time between preparation and administration of infant formulas at 1:30 and 1:25 dilution rates for full-term NBs. At a 1:30 dilution, none of the formulas analyzed exceeded 450 mOsm/kg. At a 1:25 dilution, the osmolality values of the formula for full-term NBs 1 were above the recommended by the AAP 4h after preparation both at room temperature and when refrigerated.

**Figure 2:**
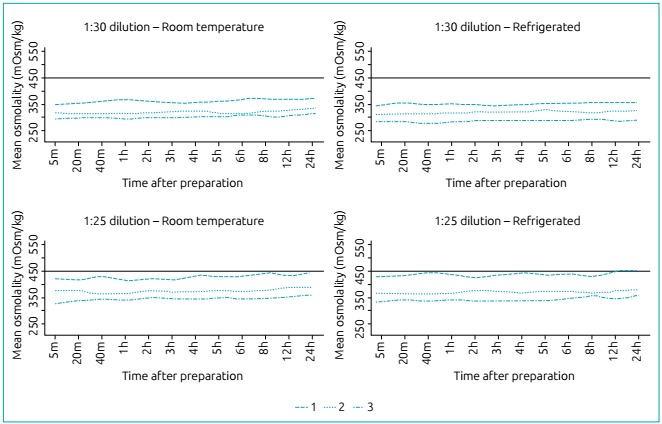
Osmolality analysis according to refrigeration and time between preparation and administration of infant formulas on the market for preterm newborns at 1:30 and 1:25 dilution rates.

Figure 2 demonstrates the osmolality curves according to refrigeration and time between preparation and administration of infant formulas for PTNBs at 1:30 and 1:25 dilution rates. The formula for PTNBs 1 exceeded the osmolality value recommended by the AAP 12h after preparation, at a 1:25 dilution when refrigerated.

## DISCUSSION

In the present study, it was possible to observe osmolality changes between the infant formulas on the market for NBs, especially when the calorie density rises (1:25 dilution).

These changes are mainly due to the type of infant formula used and the form of dilution. Among the infant formulas analyzed, the one for full-term NBs 1 showed the highest osmolality, exceeding the value recommended by the AAP 4h after preparation at a 1:25 dilution in different temperatures. We speculate that this increase might be related both to the rise in the concentration of all nutritional components and the presence of the prebiotics galacto-oligosaccharides (GOS) and fructo-oligosaccharides (FOS) in its composition. Prebiotics are non-digestible carbohydrates that stimulate the growth and/or activity of bacteria in the digestive system for the benefit of human health.^16^

Steele et al. analyzed the osmolality of 11 infant formulas, of which 7 received additives (thickeners), and found that only one more concentrated infant formula and some supplemented formulas exceeded the osmolality of 400 mOsm/kg.^11^

In clinical practice, when NBs have pathologies that do not allow a higher volume intake, a commonly used strategy is to increase the concentration of infant formula from 1:30 to 1:25.^17^ However, this practice might not be safe, due to the rise of osmolality. Another solution for this clinical issue is individualized supplementation. Thus, depending on the NB needs, which are higher the lower the gestational age, specific components can be added to the formula.^17^ According to Steele et al., fat supplementation increases the osmolality in 0.7 mOsm/kg, and carbohydrate supplementation, in 31 mOsm/kg.^11^ For Silva et al., glucose polymers and/or medium-chain triglycerides supplementation in powdered and liquid infant formulas for PTNBs provides a higher energy intake for children without exceeding the maximum recommended osmolality.^18^ This practice, however, is questionable since energy increase without the adequate proportion of amino acids can lead to a rise of fat deposition and weight, but not necessarily to compatible growth.^19^

These findings reinforce the importance of knowing the osmolality of both infant formulas and additives, and the concentration to give to PTNBs, as there are differences between them. The present study revealed that the formula for full-term NBs 1 and the formula for PTNBs 1 had their osmolality near the cut-off point (450 mOsm/kg) and its preparation and administration should follow the manufacturers’ recommendations (1:30) to prevent diseases.

Among the infant formulas analyzed in this study, none had information about the osmolality value in their label, only on the manufacturer’s website. Data on the osmolality of these products are scarce or incomplete, despite its importance in clinical practice, especially for NBs with gastrointestinal problems.^20^

Regarding the time between preparation and possible administration, the osmolality increased and exceeded the cut-off point only 12h after preparation for the formula for PTNBs 1, and 4h after preparation for the formula for full-term NBs 1. This growth in osmolality happened only at a 1:25 dilution. Evaporation and presence of prebiotics are the potential explanations for this finding, mainly because other infant formulas did not show increase associated with the time between preparation and administration.

A limitation of this study consists in the fact that the samples analyzed came from the same batch, assuming that the infant formulas complied with the standards for manufacturing quality, which did not statistically differ between batches.

We emphasize that all formulas analyzed had the following recommendation on their labels: “Consumption should be immediate. If the formula needs to be prepared in advance, it should be kept under refrigeration at a temperature below 5°C for no longer than 24 hours. Inadequate preparation, storage, and use of this product can harm the health of infants”. The manufacturer of the formula for full-term NBs 3 highlighted: “Once prepared, the formula can deteriorate quickly.”

The Collegiate Board Resolution (*Resolução de Diretoria Colegiada* - RDC) No. 50 establishes the Standards for Lactarios - dedicated areas for preparation, storage, cleaning, and sterilization of baby bottles -, which regulates the infrastructure of an appropriate lactario, but does not provide any details about the preparation of infant formulas, how long they can be kept at room temperature, let alone their recommended osmolality.^21^ Currently, the Brazilian legislation lacks protocols and guidelines for handling, storage, and administration of feeds given to NBs.

The information gaps mentioned above have led to some questions that guided the present study in its pursuit of deepening the knowledge based on scientific evidence: What is considered “immediate” consumption? Is there a safe time interval? Can the temperature influence the osmolality of these infant formulas? This study revealed that the time and temperature at which the milk was subjected after preparation did not cause the osmolality to exceed its safety cut-off point at a 1:30 dilution, only at a 1:25 dilution.

It is important to know the factors that may or may not contribute to the rise of osmolality, in order to establish safe and quality practices for NBs. This study aimed to provide evidence for the elaboration and implementation of protocols to guide procedures for handling and administration of infant formulas at neonatal intensive care units.
